# Pre-Treatment with Erythropoietin Attenuates Bilateral Renal Ischemia-Induced Cognitive Impairments

**Published:** 2018

**Authors:** Mahshid Tahamtan, Vahid Sheibani, Seyed Mostafa Shid Moosavi, Majid Asadi-Shekaari, Saeed Esmaeili-Mahani, Iraj Aghaei, Mohammad Shabani

**Affiliations:** a *Intracellular Recording Lab, Kerman Neuroscience Research Center, Neuropharmacology Institute, Kerman University of Medical Sciences, Kerman, Iran. *; b *Physiology Department, Shiraz University of Medical Sciences, Shiraz, Iran. *; c *Department of Biology, Faculty of Sciences, Shahid Bahonar University of Kerman, Kerman, Iran.*; d *Department of Neuroscience, Neuroscience Research Center, Poursina Hospital, Guilan University of Medical Sciences, Rasht, Iran.. *; e *Department of Physiology, School of Medicine, Jiroft University of Medical Sciences, Jiroft, Kerman, Iran.*

**Keywords:** Acute Kidney Injury, Bilateral Renal Ischemia, Erythropoietin, Cognitive Impairments, Memory

## Abstract

One of the most common causes of mortality in acute kidney injury is brain dysfunction. Here we investigated the possible protective effect of erythropoietin (EPO) on cognitive impairments induced by bilateral renal ischemia (BRI). Eighty male Wistar rats were allocated into 8 groups: 1, 2) Sham +V (Vehicle), 3, 4) Sham+EPO, 5, 6) BRI+V, 7, 8) BRI+EPO. The groups followed by the reperfusion periods of 24hours (24 h) and 1week (1w). EPO or saline was administrated 30 min before surgery (1000 IU/kg, i.p). The cognitive function was assessed by passive avoidance learning and Morris water maze tests. Hippocampal brain-derived neurotrophic factor (BDNF) protein expression was assessed by western blotting. BUN (blood urea nitrogen) and creatinine (Cr) concentrations were significantly increased in BRI+V group 24 h after reperfusion. BRI+V rats had just an increased level of BUN but not Cr 1w after reperfusion. EPO reversed passive avoidance learning impairments observed in BRI+V group 24 h after reperfusion. There were no significant differences in spatial and passive avoidance learning between experimental groups 1w after reperfusion and histological evaluation confirmed the behavioral data. BRI significantly decreased the BDNF protein expression in the hippocampus and EPO increased that 24 h after operation. These observations showed protective effect of EPO against cognitive dysfunctions following BRI 24 h after reperfusion through increase in BDNF protein expression.

## Introduction

 Acute kidney injury (AKI) is a common serious condition that some of critically ill patients especially in intensive care units suffer from that (1). Renal ischemia reperfusion is a leading cause of AKI (2, 3). Despite advancements in renal replacement therapy, the mortality rate during AKI has remained unchanged, which is largely due to the remote organ dysfunction (4). Accordingly, understanding the specific extrarenal manifestations of AKI would facilitate the design of therapeutic interventions to reduce morbidity and mortality after AKI. 

 Particularly, AKI can adversely affect the brain (4, 5). Cognitive changes following renal disease have been demonstrated recently. Patients with AKI commonly develop encephalopathy and altered higher mental functions (6). Hemodialysis and chronic kidney disease (CKD) patients suffer moderate to severe cognitive impairment which is dependent of the severity of CKD (7, 8). Despite of these findings, the reasons of cognitive alterations are largely unknown. Experimental evidences have established increased neuronal pyknosis, microgliosis, and inflammation in CA1 region of hippocampus which plays an important role in learning, memory and anxiety (9, 10). These evidences caused to select the hippocampus for investigation in this research.

 There are some evidences indicating the beneficial effects of EPO including anti-apoptotic, anti-inflammatory, antioxidant, and angiogenic effects (11-13). Furthermore, the expression of EPO and EPOR (erythropoietin receptor) mRNA has been showed in the hippocampus which is increased after hypoxia (14, 15). Several lines of evidences demonstrate neuroprotection effects of EPO in different animal models of brain injury such as spinal cord injury (16-18), stroke (19, 20) and autoimmune encephalomyelitis (21, 22). Recently, brain-derived neurotrophic factor (BDNF) is also known to be useful to neuronal functions and protect the CNS against a variety of brain injuries (23-25).

 Encouraged by these reports, in the present study, we first investigated the effect of AKI on cognitive performance of rats and potential neuroprotective impact of EPO to suggest a possible therapeutic intervention in clinical practice, then to determine the possible molecular mechanism responsible for AKI-induced alterations of memory function, we examined BDNF expression in the hippocampus of the experimental groups.

## Experimental

 All animal study protocols were approved by the animal care and use committee of the Kerman Medical University (Ethics Code: KNRC/92/6). Male Wistar rats (8-10 weeks old, 180-220 g) were housed under standard conditions (12/12 light dark cycle) throughout the study. 

*Experimental groups*

 In the present study, 80 male Wistar rats were randomly allocated into 8 experimental groups. The rats subjected to BRI/reperfusion were divided into four groups of n = 10, in which 0.5 mL of normal saline alone or normal saline containing EPO (1000 U/kg) was i.p. injected 30 min before the induction of renal ischemia followed by the reperfusion periods of 24 h (BRI-24 h +V group and BRI-24 h +EPO group, respectively) and 1 week (BRI-1w+V group and BRI-1w +EPO group, respectively). Sham-operated rats were also divided into four groups of n = 10 that received 0.5 mL of normal saline alone or normal saline containing EPO (1000 IU/kg) with periods equivalent to reperfusion of 24 h (sham-24 h+ V group and sham-24 h+ EPO) group, respectively) or 1 week (sham-1w +V group and sham-1w +EPO group, respectively).

*Surgery and experimental protocol*

 Each rat was anesthetized deeply by ether and the abdominal region was shaved, soaked with betadine and covered with a sterile draper, leaving only its abdomen exposed for a midline electrosurgical laparotomy. In rats subjected to renal ischemia/reperfusion, there was occlusion of the right and left renal arteries and veins for 1 h in the BRI +V group by using a non-traumatic clamp, occlusion was verified visually by a color change of the kidney to a paler shade, and reperfusion was verified by a darkening.

 In the sham +V group, all surgical procedures were performed but renal pedicles were only manipulated, and were maintained under anesthesia for the duration of the experiment. The abdominal incision was sutured at two layers by 2-0 silk. The whole surgical procedure was performed under sterile condition, and the rat was allowed to recover from the anesthesia prior returning to an individual cage. The surgical protocol was approved by the Shiraz University of Medical Sciences. 

*Treatment*

 The animals received saline (i.p.), or EPO (1,000 U/kg, single dose, (i.p) 30 min before surgery. EPO was provided as a gift from Pouyesh Darou product Company. The dose of EPO used was based on that found to be maximally effective in our previous published study (26).

*Assessment of Renal Function*

At the end of the experiments, 1 mL blood samples were collected from anesthetized rats 24h and 1w after the reperfusion period. Plasma concentrations of BUN (blood urea nitrogen) and creatinine (Cr) were used as a marker of renal function. The measurements were performed by a lab technician blind to the groups.

*Behavioral assessments*

*Passive Avoidance (PA) Learning*

This test was used to evaluate fear learning in AKI rats and the possible effect of EPO on 24 h and 1w after reperfusion. The apparatus was a shuttle-box device with dimensions of 100*25*25 which consisted of two compartments, one dark and one illuminated part separated by a guillotine door, and a stainless rod grid serving as the base. The adaptation phase was followed by a single trial in which the animals were placed in the lit arena, after 10s, the door was opened and the animal was allowed to go to the dark compartment. Then the door was closed without electric shock and after 20s, the animal was placed in the cage. If the rats did not enter the dark chamber within 60s, they were eliminated from the test and replaced with a new rat. After 30 min of adaptation, for the learning trial this procedure was repeated. A mild shock was immediately administered (0.5 mA, 1.5 s) to the rat the third time that the animal entered the dark sector. This procedure was repeated until the animal did not enter the dark box in a 60s period. The number of shocks received was recorded in this phase.

In the retention trials, 24h after training, the test was performed to evaluate memory; in this step the animal was placed in the light arena and the latency to step into the dark sector as retention time (step through latency (STL), Time in dark compartment (TDC) and number of entrance into dark sector were measured as indicators of contextual memory) was recorded to a maximum of 300s (27).

*Morris Water Maze (MWM)*

To assess spatial learning and memory, the animals were tested by a MWM as previously described (28). Briefly, the test chamber was a circular tank (140 cm diameter, 45 cm height, a black pool), surrounded by extra-tank visual cues. A visible or submerged platform (15 cm wide, 35 cm height) was placed 1.5 cm above or below the water surface. Water temperature was maintained among 21–23 °C.

Rats’ behavior was recorded with the Ethovision system. The following parameters were recorded for each rat: total distance and time spent to reach the platform in three consecutive trials, number of crosses in the correct quadrant in the retention phase, percentage of time, and distance traveled in the correct quadrant.

In the training phase, each rat underwent three blocks of trials, each comprising four trials (inter-trial interval = 30 s). In each trial, animals were placed in one of the four quadrants facing towards the maze. Each rat was given 60s to find the platform, and if they did not find it, they were put on the platform by the examiner. After 30 s, the rat was again put to the trial.

After 2 h of the last block, the rats underwent a probe trial during which the platform was removed from the tank and the number of crosses in the correct quadrant and total time spent in target quadrant was recorded and analyzed for each rat.

*Histological Assessment*

The rats were decapitated and their brains were removed 24 h and 1w after reperfusion. After tissue processing, paraffinzed brains were cut onto 6µm sections on a rotary microtome and the sections were stained with cresyl violet (Nissl staining) (29).

*Molecular experiment*

*Tissue dissection and preparation for Western blot*

For molecular experiment, the rats were allocated in sham +V, BRI +V, and BRI +EPO groups. All of the animals were anesthetized with atmosphere CO_2_ and decapitated 24 h after surgery. Their brains were rapidly extracted and placed on ice. The both whole hippocampi were freshly harvested and maintained in a microtube at −80 °C until homogenization for further western blot assay (30).

*BDNF immunoblot analysis*

Dissociated hippocampus tissues were homogenized in ice-cold buffer containing 10 mM Tris–HCl (pH 7.4), 1 mM EDTA, 0.1% SDS, 0.1% Na-deoxycholate, 1% NP-40 with protease inhibitors (1 mM phenylmethylsulfonyl fluoride, 2.5 μg/mL of leupeptin, 10 μg/mL of aprotinin) and 1 mM sodium orthovanadate (a phosphatase inhibitor). The homogenate was centrifuged at 15,000 rpm for 20 min at 4 °C. The resulting supernatant was retained as the whole cell fraction. Protein concentrations were measured using the Bradford method (Bio-Rad Laboratories, Muenchen, Germany). Equal amounts of protein were resolved electrophoretically on a 9% SDS polyacrylamide gel electrophoresis (SDS-PAGE) SDS-PAGE gel and transferred to PVDF (polyvinylidenefluoride) membranes. After blocking (overnight at 4 °C) with 5% non-fat dried milk in Tris-buffered saline with Tween 20 (blocking buffer, TBS-T, 150 mM NaCl, 20 mM Tris–HCl, pH 7.5, 0.1% Tween 20) for 2 h, at room temperature and then, the membranes were incubated overnight by a primary rabbit polyclonal antibody for BDNF (1:1000, sc-20981; Santa Cruz Biotechnology, Santa Cruz, USA) at 4 °C. After washing in TBS-T buffer (three times for 5 min each, at room temperature) the blots were incubated for 2 h at room temperature with an anti-rabbit IgG secondary antibody conjugated with horseradish peroxidase (1:15,000; GE Healthcare Bio-Sciences). Both primary and secondary antibodies were diluted in blocking buffer. The antibody-antigen complexes were revealed using the ECL system (Amersham Biosciences) and images were captured on a Gel Doc imaging system (Bio-Rad, Hercules, CA, USA), converted to a tiff file, and analyzed with Lab Work analyzing software (UVP, UK) was used to analyze the intensity of the expression. β-actin immunoblotting (antibody from Cell Signaling Technology, INC. Beverly, MA, USA; 1:1000) was used to control for loading (30).

*Statistical Analysis*

Statistical procedures were performed using the SPSS software (version 18). ANOVA followed by Tukey’s post-hoc analysis was used to compare the differences between groups. The PA learning and probe data of MWM were analyzed by one-way ANOVA. Repeated measures ANOVA was used to analyze the data of MWM task in the learning phase. 

The band density values were expressed as BDNF/β-actin ratio for each sample. The averages for different groups were compared using one-way ANOVA. All data were expressed as mean ± SEM. *P *<0.05 was considered statistically significant.

## Results

*The effect of BRI and EPO administration on plasma variables*

As demonstrated in Table 1A, 24 h after reperfusion, plasma concentrations of creatinine and urea nitrogen were significantly increased in BRI +V group compared to the other groups (*P *< 0.001). Administration of EPO counteracted these effects of BRI and reduced these plasma concentrations (*P *< 0.001).

Also in table 1B plasma concentrations of creatinine and urea nitrogen has been showed 1w after operation. Urea nitrogen was significantly increased in BRI +V group (*P *< 0.05 versus sham +V) and administration of EPO reduced that (*P *< 0.01), but there are no significant difference in creatinine level between the groups (*P *> 0.05).

*The effect of BRI and EPO administration on PA learning 24 h after reperfusion*

**Figure 1:**
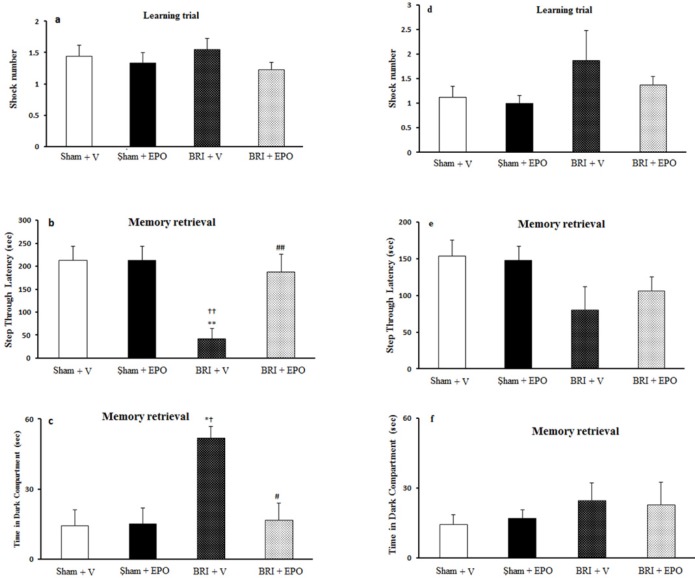
The BRI effect of and treatment with EPO on the fear learning in passive avoidance learning paradigm 24h and 1w after reperfusion. (a) number of shocks received in the training day was not altered amongst four groups, which indicates that fear learning is not changed between four groups of study. (b) decreased step through latency (STL) in BRI + V animals shows an impaired fear memory. Administration of EPO reversed these deficits. (c) time in dark compartment was significantly increased in BRI + V group which demonstrate context-dependent learning impairment in BRI + V rats. Administration of EPO reduced significantly this effect of BRI on fear memory. (d) there was no significant alteration in the number of shocks received in the training, which indicates that fear learning is not altered amongst experimental groups. (e) step through latency (STL) was not altered between four groups which implies that fear memory is not altered among four groups of study. (f) time in dark compartment was not changed significantly between the groups. **P < 0.01, as compared to sham +V group; ††*P *< 0.01 as compared to sham + EPO group; ## *P *= 0.01 as compared to BRI +V group. # *P *< 0.05 as compared to BRI + V group, *†*P *< 0.05 compared to sham +V and sham + EPO

There was no significant difference in the number of shocks received among the four groups (*P *> 0.05) (Figure 1a).

RI +V rats had impairment in memory retrieval compared to the other three groups (*P *< 0.01, ANOVA); administration of EPO reversed this impairment (Figure.1b).

The time in the dark compartment (TDC) was altered in the BRI +V group compared to the BRI + EPO group (*P*< 0.05, ANOVA). EPO counteracted this effect of BRI on PA learning (Figure 1c).

**Table 1:** The effect of BRI and EPO administration on plasma variables 24 h after surgery.

**Variables**	**Plasma creatinine** **(mg/dL)**	**Plasma urea nitrogen** **(mg/dL)**
Groups		
Sham + V	0.68 ± 0.03	37.8 ± 3.5
Sham + EPO	0.71 ± 0.04	38 ± 3.3
BRI + V	1.5 ± 0.19^*** †††^	192.1 ± 22.06^*** †††^
BRI + EPO	0.67 ± 0.04	42.4 ± 1.7

**Table 2:** The effect of BRI and EPO administration on plasma variables 1 week after surgery.

**Variables**	**Plasma creatinine** **(mg/dL)**	**Plasma urea nitrogen** **(mg/dL)**
Groups		
Sham + V	0.62 ± 0.04	35.6 ± 4.4
Sham + EPO	0.60 ± 0.02	31.6 ± 3.6
BRI + V	0.60 ± 0.05	58.8 ± 6.6*, ††
BRI + EPO	0.57 ± 0.15	33.16 ± 3.9

Values are means ± SEM at 24 h (1 A) and 1 week (1.B) after bilateral renal arteries and veins obstruction in different groups.

*** *P *< 0.001 versus Sham +V,

††† *P *< 0.001 versus Sham + EPO and BRI + EPO.

* *P *< 0.05 versus sham +V,

†† *P *< 0.01 versus Sham + EPO and BRI + EPO.

**Figure 2:**
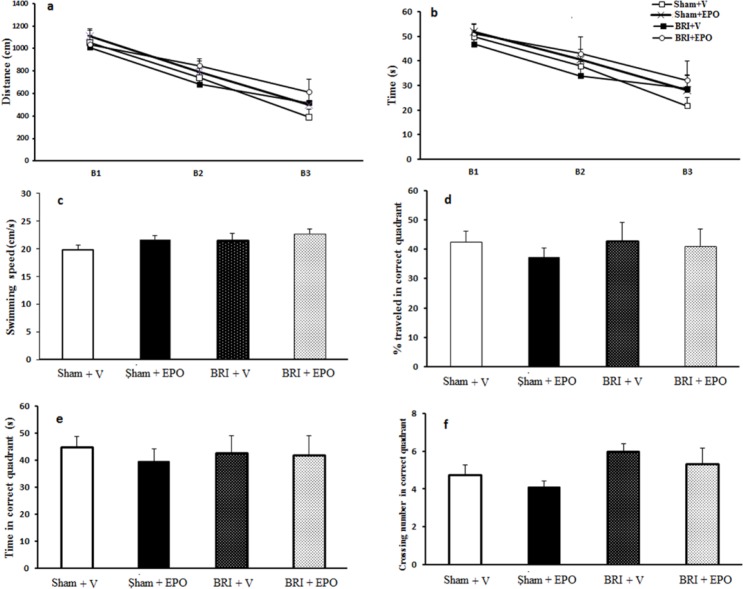
The effect of BRI on spatial learning and memory. (a) and (b) a reduced time and latency to reach the platform indicates spatial acquisition in all rats (*P *< 0.05). There was no significant difference observed in the probe trial parameters (Figure. 2 c_f) measured among the four groups of study (*P* > 0.05

**Figure 3:**
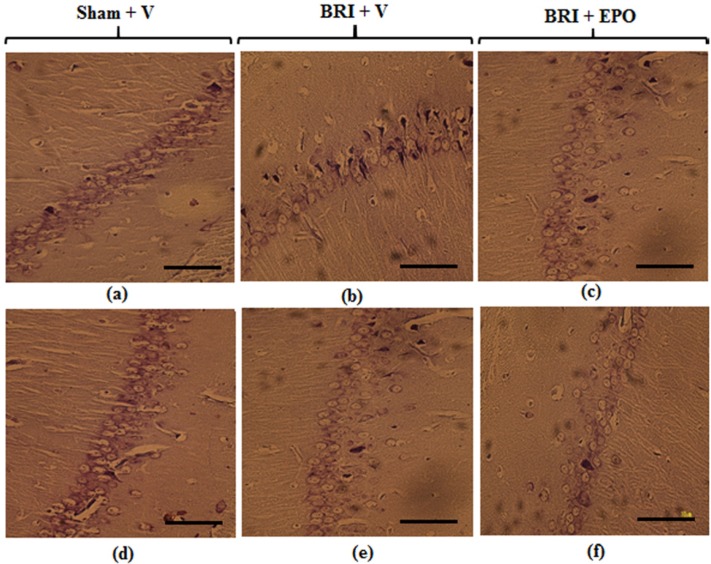
Neurons of the CA1 region of hippocampus 24 h and 1w after reperfusion. Most neurons of the hippocampus in sham + V group have normal morphology (a) but for the BRI + V group, several degenerated cells can be seen with shrinkage nuclei and dark cytoplasm (b). EPO could attenuate degenerative changes induced by BRI (c). Normal morphology of pyramidal neurons was observed in all experimental groups (d_f) (Scale bars = 50 μm

**Figure 4:**
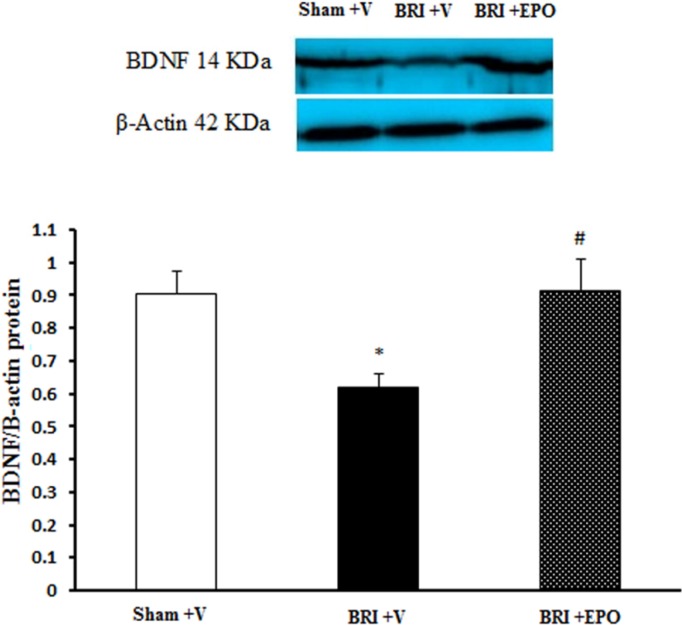
The Effect of BRI and EPO on the BDNF protein levels in the hippocampus of rats. BRI + V decreased the BDNF protein levels in the hippocampus of rats. Meanwhile EPO improved this deficit. Each value point in the graphs represents the mean ± S.E.M (5 rats/group). β-Actin was used as an internal control. *P < 0.05 as compared to sham group, ^#^*P *< 0.05 as compared to BRI + V group

*The Effect of BRI + V and EPO administration on PA learning 1 week after reperfusion*

No significant alteration in the parameters measured was observed amongst the four groups of study 1 week after reperfusion (*P *> 0.05) (Figure 1d_f).

*The effect of BRI and EPO administration on spatial learning*

Figure 2a and b shows the results of the learning trial. Repeated measures of analysis of variance showed a reduction in escape latencies at the block effect in all rats and the swimming distance to locate the platform across blocks of trials, indicating spatial acquisition (block 3 compared to block 1 for all groups, at least *P *< 0.05). All treated rats, sham + V, and the BRI + V groups progressively took less time to locate the hidden platform over the course of the 12 trials during the training ----period. Spatial learning in the MWM task was not altered in BRI + V rats compared to sham +V, sham + EPO or BRI + EPO rats (*P *> 0.05, (Figure 2a and b). There were no significant differences in the swimming speeds (Figure 2c) between groups in all periods, indicating that the swimming speed did not have influence in the latencies. 

There was no significant difference observed in the probe trial parameters (Figure 2d_f) measured among the four groups of study.

*Histological evaluation*

As Figure 3 (a-c) demonstrates 24 h after reperfusion: Light microscopic study of CA1 region of hippocampus sections showed normal morphology in pyramidal cells of sham + V group. Histologic samples of sham + EPO group also did not show any morphologic changes compared to sham (data have not been shown).

The CA1 of hippocampus sections in BRI +V group showed degenerative changes including shrunken nuclei and dark cytoplasm. Meanwhile, EPO could protect neurons from morphological alterations 24 h after induction of BRI and attenuated degenerative changes in pyramidal neurons of hippocampus region.

One week after reperfusion: Normal morphology of pyramidal neurons was observed in CA1 region of hippocampus in sham + V group. No significant degenerative changes were detected in BRI + V and BRI + EPO groups (Figure 3 d-f).

*The effect of BRI and EPO administration on BDNF protein expression*

Then, we examined whether there is a potential molecular mechanism that underlies the memory impairments. We evaluated the expression of BDNF 24 h after reperfusion, because the BRI -induced memory dysfunction was observed just in this time point. Since there was no significant difference between sham and sham + EPO groups in the behavioral data, the sham + EPO group did not assess in the molecular experiment.

As shown in Figure 4 the lowest level of BDNF protein (0.61 ± 0.03) was detected in the hippocampus of BRI + V rats and statistically significant difference was observed as compared to sham + V (0.9 ± 0.06; *P *< 0.05) and BRI + EPO rats (0.91 ± 0.09; *P *< 0.05) groups (Figure. 4). The current data showed that significant decrease in BDNF protein expression in BRI + V group was inhibited by administration of EPO.

## Discussion

The goals of this study were to assess 1) the possible learning and memory disabilities associating with BRI (as an animal model of AKI) and 2) the possible neuroprotection effect of EPO on brain dysfunction induced by BRI in two different time points: short-term (24 h after reperfusion) and long-term (1w after reperfusion). 

Our results demonstrated that BRI leads to learning and memory impairments 24 h after reperfusion. Also, we observed no significant memory disabilities in BRI + V group compared to the other groups 1w after operation.

Most of the mortality caused by AKI is due to extrarenal organ dysfunction. Experimental evidence has established that AKI leads to brain inflammation (10, 31). Liu *et al* observed that AKI led to both soluble and cellular inflammation in the brain, with the hippocampus being the main target (10). Also, they found that mice with AKI showed striking cellular abnormalities, microgliosis and increased neuronal pyknosis in the hippocampus (10). On account of the high sensitivity of hippocampus in response to AKI, cognitive dysfunctions can be proposed following AKI. 

Additionally, researchers demonstrated a graded relation between estimated glomerular filtration rate level (24) and cognitive function (32-34). In patients undergoing dialysis, cognitive impairments are seen more frequently (7, 8). When the disease progresses, disorientation, defects in attention span and memory becomes manifest (35). 

Pharmacological correction of brain dysfunction associated with AKI is clinically important, which is a reason for studying ischemia and the identifying new treatment strategies affording neuroprotection. In the current study, EPO showed a neuroprotective function against learning and memory deficits in ischemic rats 24 h after reperfusion. 

EPO is a glycopeptide that not only plays a key role in erythrocyte production stimulation in the bone marrow, but also known as a neuroprotective agent offers potential (36). EPO has multiple protective effects, such as antioxidant, anti-inflammatory, angiogenic and antiapoptotic effects (37). Since the significant effects of AKI on brain pathologies has become increasingly clear which involves inflammation; therefore, it seems that EPO may exert neuroprotective effects against BRI -induced impairments. 

The data of the present study clearly demonstrate that BRI (as an animal model of AKI) leads to PA learning impairment 24 after reperfusion. EPO treated rats had STL and also TDC similar to the sham +V group. Therefore, EPO showed a promising effect against learning and memory impairments induced by BRI 24 h after reperfusion. Passive avoidance test also was performed 1 week after reperfusion and data obtained with this test indicated no significant differences between measured parameters between the groups. Our findings are consistent with previous studies reporting cognitive impairments in renal diseases (as mentioned above). 

In the current study, the spatial memory was evaluated using a Morris water maze 1 week after reperfusion and data indicated no significant differences in memory amongst the groups.

As mentioned above, most notably, we discovered that 1 week after BRI, there are no significant cognitive deficits. There are some potential explanations for these findings. First, we could argue through the comparison of plasma variables 24 h and 1w after reperfusion. Our data clearly demonstrate that 60 min bilateral renal ischemia reperfusion caused significant increase in the plasma concentrations of both BUN and Cr 24 h after ischemia, indicating a significant level of renal dysfunction. One week after ischemia, although BUN level is significantly more than the other groups but there is no significant difference in plasma concentration of creatinine between experimental groups. Since the change in creatinine is clinically and pathologically an important indicator of AKI, it can be suggested that the renal function recovered to some extent 1 week after BRI and it is likely the factors involved in the pathogenesis of the kidney tissue injury during I/R reduced.

Second, in this study, histological results also demonstrated that 24 h after reperfusion CA1 of hippocampus sections in BRI + V group showed severe injury and EPO could protect against renal ischemia and decreased degeneration compared to BRI + V group. CA1 region is related with memory and deficits of these neurons leads to behavioral dysfunctions assessed by laboratory tests.

In contrast to this, one week after reperfusion no significant degenerative changes were observed in CA1 region of hippocampus sections in all experimental groups.

Shid Moosavi *et al* have previously indicated that 24 h of unilateral ureteral obstruction leads to oxidative stress in the obstructed kidney through not only enhanced production of reactive oxygen species (ROS) but also decreased ability of antioxidant defense system (38). Third, in the light of these findings, since oxidative stress observed at early hours after release of obstruction, we can explain severe damages at 24 h compared to 1w. Our results showed that EPO as an antioxidant agent could prevent impairments has been evented at first 24 h after BRI. 

The protective effect of EPO on renal and cerebral ischemia, separately has been shown in various studies (39-42). For instance, Tazangi *et al* reported that the treatment with EPO can improve the memory loss and synaptic plasticity defect in the rat model of Alzheimer’s disease (43). Also the recovery of spatial memory in brain-lesioned animals following EPO administration has been observed (44). EPO can improve nerve function by the modulation of transient neural plasticity mechanisms (44).

In the current study molecular assay was performed at 24 h after reperfusion (because we did not observe learning and memory impairment 1w after reperfusion, therefore did not examine in this time point). The results of our molecular assay indicated that EPO administration improved decrease in BDNF protein level in hippocampus region which was induced by BRI 24 h after reperfusion. Several lines of evidences demonstrated contributions of BDNF in cognitive functions, particularly in memory acquisition and consolidation (24, 45, 46). The present study for the first time provides clear evidences that a single dose of EPO administration 30 min before renal ischemia can confer marked behavioral, histological and molecular protection 24 h after reperfusion. Protection induced by EPO may be mediated by several mechanisms: 1) EPO treatment can increase generation of neurotrophic factors and induce long term changes in cognition (39). 2) EPO can reduce lipid peroxidation and support defense systems which are important insults after renal ischemia (47, 48). 3) It has been previously shown that impairments in the cholinergic pathways of the brain may be responsible for the cognitive deficits (49).

EPO leads to increased cholinergic function and as its result, improvement of cognitive deficits (50, 51). We just summarize some possible mechanisms of EPO but further studies are needed to clarify the exact mechanisms of EPO neuroprotection in a BRI model of cognitive impairments.

In conclusions, results from our experiments showed that EPO when administered as a single dose 30 min before BRI, improved deficits 24 h after reperfusion. One of the potential mechanisms involved in this protection may be through increase in BDNF protein expression. Also, we observed that there are no significant behavioral abnormalities in BRI group 1w after ischemia.
